# Evaluation of the solar-powered Silver Bullet 2.1 (Lumin 8) light trap for sampling malaria vectors in western Kenya

**DOI:** 10.1186/s12936-023-04707-y

**Published:** 2023-09-16

**Authors:** Oscar Mbare, Margaret Mendi Njoroge, Fedinand Ong’wen, Tullu Bukhari, Ulrike Fillinger

**Affiliations:** International Centre of Insect Physiology and Ecology, Human Health Theme, 30772 – 00100, Nairobi, Kenya

**Keywords:** Light traps, *Anopheles*, Culicines, Trap efficiency, UV, Wavelength

## Abstract

**Background:**

Centers for Disease Control and Prevention (CDC) light traps are widely used for sampling mosquitoes. However, this trap, manufactured in the USA, poses challenges for use in sub-Saharan Africa due to procurement costs and shipping time. Traps that are equally efficient than the CDC light trap, but which are amenable for use in remote African settings and made in Africa, are desirable to improve local vector surveillance. This study evaluated a novel solar-powered light trap made in South Africa (Silver Bullet trap; SB), for its efficiency in malaria vector sampling in western Kenya.

**Methods:**

Large cage (173.7 m^3^) experiments and field evaluations were conducted to compare the CDC-incandescent light trap (CDC-iLT), CDC-UV fluorescent tube light trap (CDC-UV), SB with white diodes (SB-White) and SB with UV diodes (SB-UV) for sampling *Anopheles* mosquitoes. Field assessments were done indoors and outdoors following a Latin square design. The wavelengths and absolute spectral irradiance of traps were compared using spectrometry.

**Results:**

The odds of catching a released *Anopheles* in the large cage experiments with the SB-UV under ambient conditions in the presence of a CDC-iLT in the same system was three times higher than what would have been expected when the two traps were equally attractive (odds ratio (OR) 3.2, 95% confidence interval CI 2.8–3.7, P < 0.01)). However, when the white light diode was used in the SB trap, it could not compete with the CDC-iLT (OR 0.56, 95% CI 0.48–0.66, p < 0.01) when the two traps were provided as choices in a closed system. In the field, the CDC and Silver Bullet traps were equally effective in mosquito sampling. Irrespective of manufacturer, traps emitting UV light performed better than white or incandescent light for indoor sampling, collecting two times more *Anopheles funestus *sensu lato* (s.l.)* (RR 2.5; 95% CI 1.7–3.8) and *Anopheles gambiae s.l.* (RR 2.5; 95% 1.7–3.6). Outdoor collections were lower than indoor collections and similar for all light sources and traps.

**Conclusions:**

The solar-powered SB trap compared well with the CDC trap in the field and presents a promising new surveillance device especially when charging on mains electricity is challenging in remote settings.

## Background

Malaria transmission is determined by the interaction between the malaria parasite, mosquito vector and human host [1], and the spatial and temporal variations in mosquito abundance determines transmission patterns and intensity [2]. Systematic mosquito surveillance is critical in any mosquito-transmitted disease control program to determine the composition and abundance of potential vector species over space and time [3, 4]. Mosquito surveillance is largely done by collecting adult vectors using traps that have proven efficient in sampling the target vector species and the physiological stage pursued for collection [5, 6]. The human landing catch (HLC) has been the historical gold standard for sampling host-seeking malaria vectors since it provides a direct and reliable estimate of the host-vector contact which is required to estimate disease transmission intensity [7]. However, the use of HLC raises serious ethical concerns as it exposes the human collectors to potential infectious mosquito bites [8]. HLC is also labour-intensive, difficult to supervise and standardize, and is subject to significant collector bias [9, 10], hence limiting its use in routine mosquito surveillance [11].

Several alternative mechanical sampling tools and techniques including traps that use olfactory (e.g. carbon dioxide) [12] or visual (e.g. light) [13] cues to lure mosquitoes are available for sampling host-seeking malaria vectors. However, every sampling tool performs differently, none providing absolute numbers and hence comparing collections made with different tools is not straight forward [14, 15]. Light trapping is an important tool for monitoring mosquito populations by tracking the presence and abundance of the vector species [16, 17]. The CDC miniature light trap, developed by the Centers for Disease Control (CDC, USA), is a portable sampling device for exposure-free collection of mosquitoes and sand flies [18] which was introduced in the 1960s [19], and has since become a standard mosquito collection equipment for both surveillance and interpretation of the effects of vector control interventions worldwide [20–23]. Several studies have shown that it provides comparable estimates of vector densities to the HLC in a range of indoor settings, and its wide use provides the opportunity to compare vector densities over space and time [24, 25]. The trap is usually placed close to a blood-host for the target vector species and the mosquitoes are attracted to the host-odours and at closer range to the light. A downward suction of the air by a battery-powered fan ensures that mosquitoes in close-range are pulled into a collection bag [26]. Whilst CDC light traps are available with fluorescent blue-black light tubes [27] and more recently with diodes of different colours [28], the most widely used CDC miniature light trap in vector surveillance programmes in Africa typically contains a 4–6 watts incandescent light bulb and is run with support of 6 V or 12 V batteries [29–31].

The CDC miniature light trap is relatively easy to transport and deploy since all parts are collapsible and lightweight [19]. However, the need for batteries and their daily recharge to power the traps present a major challenge in the electricity-deficient rural areas in sub-Saharan Africa [32]. Furthermore, all CDC light traps in the market are currently manufactured by two major suppliers in the USA [18, 33]. Thus, African vector surveillance and control programmes, as well as research programmes, need to budget significant funds for the shipment of the traps and plan for extended waiting times from order to delivery in the study locations. Also, the lightweight construction of the CDC trap, whilst generally desirable, affects its sturdiness and durability when used in remote African settings, resulting in frequent down-times of the traps. It is against this background that the new solar-powered Silver Bullet 2.1 mosquito trap was developed by the South African manufacturer Lumin 8 [34]. This novel trap is robustly built and has a built-in/integrated battery that can be charged by either solar energy, mains electricity by means of an international power adapter, or through a vehicle auxiliary power cable (cigarette lighter socket). To lure biting disease vectors into the trap, this novel trap is fitted with a configurable cluster of differently coloured light emitting diodes (LEDs) [34]. The aim of this study was to assess the efficiency of the new Silver Bullet trap (SB) in sampling malaria mosquitoes in western Kenya. The performance of the SB trap in sampling these mosquitoes was compared against the CDC miniature incandescent light trap to assess its suitability as a vector surveillance tool.

## Methods

### Study area

Large cage experiments were implemented at the International Centre of Insect Physiology and Ecology-Thomas Odhiambo Campus (*icipe*-TOC) in Mbita, Homa Bay County, western Kenya (0°25′56.16" South, 34°12′26.95" East). The cage enclosure, referred to as semi-field system, was rectangular with two long sides, measuring 10.8 m and two shorter sides measuring 6.7 m wide. The maximum height of the system was 2.4 m (volume, 173.7 m^3^). The walls and the ceiling of the system were screened with black fibreglass netting gauze (1.7 mm × 1.5 mm). A gable roof above the ceiling made from translucent polycarbonate protected it from rain. The floor was covered with sand to a depth of 30 cm.

Field assessments were conducted in Burudu village (0°15′34.43" North; 34° 5′2.21" East) in Nangosia Location, Samia sub-County, Busia County in the highly malaria endemic Lake Victoria region [35], western Kenya. Four houses were purposively selected from the village, based on house characteristics. The houses were single roomed (approximately 6.25 m^2^), had grass-thatched roofs, open gaps around the roofs’ eaves, mud walls and un-stabilized earthen floors. Each of the selected houses had at least three cattle sleeping in the homestead between 5–15 m away from the house at night.

## Traps

### CDC light traps

**Fig. 1:**
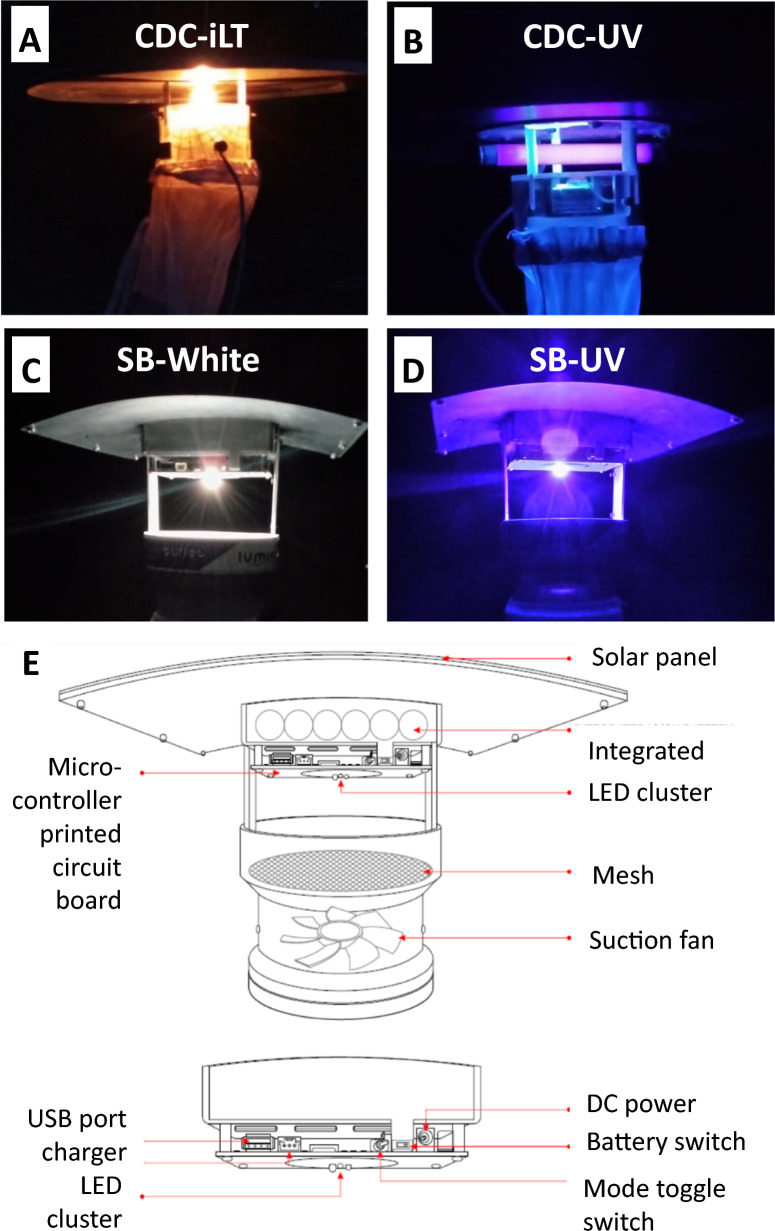
Images showing light emitted at night by the **A** CDC-iLT, **B** CDC-UV, **C** SB-White and **D** SB-UV traps, and **E** schematic sketch of parts of the Silver Bullet trap

The standard CDC Miniature Incandescent Light Trap Model 1012 (CDC-iLT) and the CDC Downdraft Blacklight (UV) Trap Model 912 (CDC-UV) manufactured by John W Hock, Gainsville, USA were used for experiments. The CDC-iLT served throughout as the reference trap. Detailed descriptions of the traps are provided by the manufacturer [36]. Briefly, the CDC-iLT was fitted with a 4-Watt incandescent light bulb and powered by a 6 V battery while the CDC-UV was fitted with a 4-Watt blue-black fluorescent tube and powered by a 12 V battery (Fig. 1). The CDC-UV light emits black (UV) light while the CDC-iLT emits incandescent light. These traps were made of acrylic to protect the motor, fan, and the light sources and a black rain shield positioned at the top of the trap when assembled. Both traps used downdraft fans to create air suction to pull and keep insects in the traps’ collection bags [36].

### Silver Bullet light traps

The Silver Bullet 2.1 trap was made of polyvinyl chloride (PVC) and aluminium providing a protective casing for all parts, hence preventing breakage during transport and accidental falls in the field. An indestructible polycrystalline 12-Watt solar panel at the top of the trap charged the trap’s lithium-ion battery pack when placed in the sun. The trap also had a plug-in mains adapter that could be used to charge it. Three light emitting diodes (LEDs) that emit UV, white and red lights were fitted on the trap and a programmable microcontroller controlled the LED output. In addition, the traps had a USB port charger, which can be used for charging of mobile phones and tablets. A downdraft suction fan pulled insects into the trap.

The Silver Bullet trap was tested in two settings based on the colour of the LED light. The trap was programmed to either emit light with the UV LED (SB-UV trap) or white LED (SB-White trap).

### Large cage (semi-field) experiments

Insectary-reared adult *Anopheles gambiae *sensu stricto (*s.s*.) (Mbita strain) and *Anopheles arabiensis* (Mwea strain) were obtained from the *icipe*-TOC mosquito insectary where they were reared following standard procedures [37]. The mosquitoes had never fed on blood when they were selected for experiments. Seven hours prior to the start of experiments, 80 female *An. gambiae s.s.* and 80 female *An. arabiensis*, between 3 and 5 days old, were selected from insectary cages for use in experiments. The two *Anopheles* species were introduced into separate paper cups for holding. The top of each paper cup was covered with a cotton netting material. During their time in the paper cups, mosquitoes were provided with only water on moistened towel placed on top of the netting material. Two hours prior to the start of experiments, the mosquitoes in the holding cups were dusted with two different fluorescent colour dyes to distinguish the two species in trap catches [38].

Experiments in the semi-field system were used to test whether there is any difference between the SB trap and CDC trap in collecting the insectary-reared mosquitoes. These experiments allowed for direct comparison in the efficacy of the two traps to recollect the released insectary-reared mosquitoes. During experiments two traps were placed at opposite corners of one of the shorter walls of the rectangular semi-field system in a competing set-up. On each experimental night, the opposing shorter wall of the system served as the release point of the mosquitoes from their holding cups. To release mosquitoes for experiments, the netting on top of the holding cups was removed to allow mosquitoes to freely leave the cup and orient towards the traps in the system. Bias that could occur due to the position of the two traps in a semi-field system was minimized by systematically rotating the shorter side of the system where the traps were placed.

**Fig. 2:**
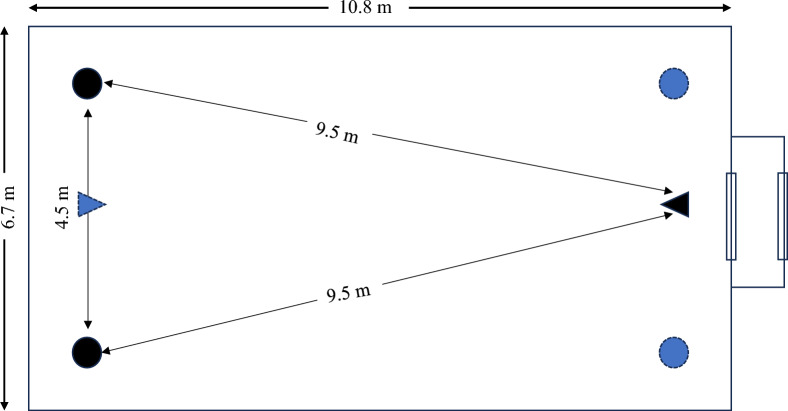
Schematic diagram of trap positions and release positions of mosquitoes in the semi-field systems. Traps positions are shown in circles while mosquito release points in triangles. Colour codes show corresponding trap positions and mosquito release points

Two semi-field systems were used concurrently. One system served as the reference where two CDC-iLTs presenting two equal choices were set up. In a well-calibrated system, this equal choice set-up will result in a balanced response of the released mosquitoes towards the two traps [39]. In the test set up, in the second system, choices were provided between: (1) CDC-iLT and SB-White; or (2) CDC-iLT and SB-UV. The two traps in a semi-field system were 4.5 m apart and equidistant from the release point of the mosquitoes which was 9.5 m away from the traps (Fig. 2). At all times, the light traps were always supplemented with an odour cue simulating a host. The cue was artificial carbon dioxide produced by mixing 250 g of molasses (Mumias Sugar Company Ltd, Kenya), 17.5 g dry yeast (Angel^®^ Yeast Company Ltd, China) and 2 l of water. The carbon dioxide was released through a small pipe close to trap entry point [40], and a fresh mixture was used for each experimental night and trap. These experiments were repeated over 16 rounds. Each experimental round started at 18.00 h when mosquitoes were released in the semi-field systems and the number of mosquitoes recollected in each trap was recorded the next morning at 07.00 h when experiments were stopped. Thereafter, all mosquitoes remaining in the semi-field system were aspirated using a motorized aspirator before the start of the next round of experiment.

### Field evaluations

A randomized 4 × 4 Latin square design was used to assess the traps’ effectiveness in collecting wild mosquitoes. Four houses located approximately 500 m apart were selected. The four traps, namely a CDC-iLT, a CDC-UV, a SB-White and a SB-UV, were randomly allocated to one of the houses on each trap night. The experiment was conducted over 64 nights comprising of 16 trap nights indoors and 16 trap nights outdoors for each trap. Indoor and outdoor collections were done independently during alternating nights. When traps were placed indoors, they were hung adjacent to the foot end of a person sleeping under an insecticide-treated bed net (Olyset Net, Sumitomo Chemical), while outdoors, traps were hung within 1 m of cattle resting place (1–3 animals) in the households. The traps were switched on at 18.00 h and stopped the following morning at 07.00 h. These field evaluations were conducted during the rainy season (May–June).

All field caught *Anopheles* were identified using the morphological key by Coetzee [41]. All members of the *An. gambiae *sensu lato (*s.l.*), and *Anopheles funestus s.l.*, were further identified to species level using polymerase chain reaction (PCR) [42]. Culicinae were classified into genera.

### Light quality measurements

Behavioural differences in attracting mosquitoes to traps might be associated with differences in light quality [43]. Spectrometry was used to establish wavelength and intensity of light emitted from the traps [44, 45]. All light measurements were done in a dark indoor arena (8.4 m × 6.3 m × 2.7 m) with all walls covered in black polyester blackout fabric. For the measurements, traps were suspended with a tripod 1.5 m from ground level. To estimate the light stimuli that might be perceived by an advancing mosquito, the absolute spectral irradiance (intensity of light at each wavelength) was measured directly at the point of emission and 1 m away from the trap. All measurements were replicated three times and averaged. There were no other objects in the arena except the traps, tripods and spectrometry kit. These absolute spectral reflectance measurements (wavelength and intensity) were taken using a solarization resistance optical fibre (QP400-025-SR-BX, 400 μm) connected to a modular spectrometer (Flame UV–VIS, 200–850 nm), through a spectralon cosine corrector (CC-3-UV-S, 180 °C). These were coupled with a UV-Vis-NIR radiometrically calibrated light source (DH-3P-BAL-CAL, 230 nm–2.5 μm). The data was recorded using the Oceanview spectrometry software (version 1.6.7) in μW/cm^2^/nm. All equipment and software were supplied by Ocean Insight, Florida, US. Light spectra were plotted in excel with averages of absolute intensity measurements at each wavelength [46].

### Data analysis

Experimental data were analysed in R statistical software version 3.5.1 [47]. Semi-field data were analysed with generalized linear models with a binomial distribution and logit link function fitted to compare the proportion of female *An. gambiae s.l.* collected in the test traps out of all mosquitoes trapped between the two equal choice reference experiment and the two different choice experiment. Overall response of released mosquitoes was explored by comparing the proportion of released females recollected with both traps in the two semi-field systems out of all released. The experimental set ups; CDC-iLT vs CDC-iLT (reference experiment), CDC-iLT vs SB-white (test experiment 1) or CDC-iLT vs SB-UV (test experiment 2) were included in the model as fixed factors with the equal choice experiment as the reference [48]. The aim of this analysis was to investigate potential preferences for one trap over the other. The hypothesis of this choice bioassay is that when two CDC-iLTs are presented as equal choices in a semi-field system, the mosquito response towards these choices is similar with odds of success of 1:1. It is expected that when two different traps are presented with one being more attractive to mosquitoes, a statistically significant diversion from this reference would be observed. Odds ratios (OR) and their associated 95% confidence intervals (CI) are reported from the model outputs. Generalized estimating equations (GEE) were used to analyse the field data. Mosquito count data were fitted a Poisson distribution with a log link function and exchangeable correlation matrix. The trap type (CDC, SB) and colour emitted by trap as well as the interaction of both were included in the model as fixed factors while the household identifier was included as repeated measure. Light from the CDC-iLT and SB-white was described as white light while that from the CDC-UV and SB-UV traps as UV light. Mean proportions and mean counts and their associated 95% CIs were estimated based on model parameter estimates. The rate ratios (RR) and associated 95% CI were reported. The analyses were separated for indoor and outdoor mosquito catches and by mosquito species. Since *Anopheles coustani* and *Anopheles pharoensis* were caught in small numbers, catches for each trap were pooled as ‘other *Anopheles*’ for analysis. Similarly, all trapped male *Anopheles* were pooled for analysis. Female and male culicine mosquitoes were also collected in small numbers and thus all genera pooled separately for the sexes for analysis.

## Results

### Semi-field experiments

Trapping efficiency across traps was similar for *An. gambiae s.s.* and *An. arabiensis* (p = 0.6), therefore the collections of the two species per trap were pooled for the presented analysis.

Despite the presence of light and odour cues in the semi-field system, not all female mosquitoes released were actually trapped. Only around 54–56% of released mosquitoes were recovered with two traps when either two CDC-iLT were simultaneously used or when CDC-iLT were paired with a SB-UV trap (Table 1). A slightly lower proportion was recaptured when the CDC-iLT was paired with the SB-White trap. An explanation for the latter can be found in the trapping efficiency of the SB-White trap when competing with a CDC-iLT in choice tests. The odds of trapping a host-seeking female *Anopheles* with the SB-White light test trap in the presence of a CDC-iLT was 1.8-fold lower compared to the reference experiment with two equal CDC-iLTs (Table 1). However, when the UV setting was used on the SB trap, the odds of trapping an *Anopheles* in this test trap out of all trapped was 3.2 fold higher than in the reference experiment.

**Table 1:** Efficiency of trapping released female *Anopheles* mosquitoes using choice tests with CDC and SB light traps in semi-field systems

Choice experiment	Reference trap	Test trap	Mean proportion (95% CI)	Odds ratio (95% CI)	p-value
Comparing proportion of *Anopheles* trapped with both traps out of all released					
Equal choice	CDC-iLT	CDC-iLT	0.54 (0.52–0.56)	1	
Test 1	CDC-iLT	SB-White	0.42 (0.40–0.44)	0.62 (0.56–0.69)	0.002
Test 2	CDC-iLT	SB-UV	0.56 (0.54–0.58)	1.08 (0.97–1.20)	0.156
Comparing proportion of *Anopheles* trapped in test trap out of all trapped					
Equal choice	CDC-iLT	CDC-iLT	0.49 (0.46–0.51)	1	
Test 1	CDC-iLT	SB-White	0.35 (0.32–0.38)	0.56 (0.48–0.66)	0.008
Test 2	CDC-iLT	SB-UV	0.75 (0.73–0.77)	3.20 (2.75–3.74)	0.001

Experiments were repeated over 16 rounds

### Field evaluation

Over the 64 trap nights, a total of 7,356 mosquitoes were trapped, 87% (n = 6,371) were female while 13% (n = 985) were male. Sixty-four percent of the female mosquitoes were *Anopheles* (n = 4,099) and 36% were culicine mosquitoes (n = 2,272). Of the female *Anopheles* trapped indoors, 60.7% (n = 1,951) were *An. funestus s.l.*, 38.1% (n = 1,222) *An. gambiae s.l.*, 1.2% (n = 38) *An. coustani*. Outdoor collections were much lower in numbers, with *An. funestus s.l.* also predominant but only constituting 42% (n = 373) of the total female *Anopheles* collected. *Anopheles gambiae s.l.* (28.8%, n = 256) was the second-most abundant vector outdoors followed by *An. coustani.* (27.8%, n = 247) and *An. pharoensis* (1.4%, n = 12).

All females of the *An. funestus s.l.* and *An. gambiae s.l.* species complexes were analysed by PCR for species identification. For *An. funestus s.l.*, 99.8% (n = 1,947) of indoor and 92.8% (n = 346) of outdoor collections were *An. funestus s.s*. *Anopheles rivulorum* was collected in small numbers exclusively outdoors (n = 24). The remaining *An. funestus s.l.* trapped indoor (n = 4) and outdoor (n = 3) did not amplify.

The species composition of the *An. gambiae* complex was reverted in the indoor and outdoor environment. Indoors, *An. gambiae s.s.* predominated with 70.7% of the specimen collected (n = 864); *An. arabiensis* representing only 29.3% (n = 358). Outdoors, *An. gambiae s.s.* was only trapped in small numbers (7.4%, n = 19) whilst *An. arabiensis* proportionally predominated the catch (92.6%, n = 237) though still fewer than indoors in absolute numbers.

**Fig. 3:**
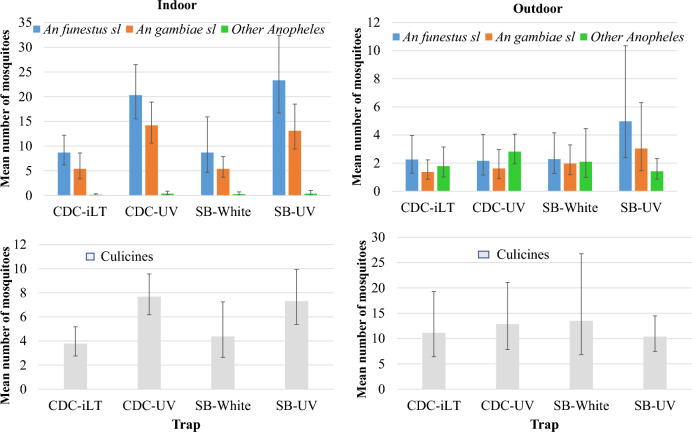
Mean number (95% confidence intervals) of female *Anopheles* and culicine mosquitoes trapped with different trap types in the indoor and outdoor environment

Overall, the estimated mean number of primary malaria vectors (*An. funestus s.l.* and *An. gambiae s.l.*) was relatively high in the selected field site with approximately 25 (95% CI 20–31) female specimen per trap night indoors. Both the CDC and SB traps performed equally under natural field conditions in trapping *Anopheles* and culicine mosquitoes. Not the trap type, but the quality of light was significantly associated with the number trapped indoors. The use of UV light in indoor traps was over two times more efficient in attracting malaria vectors from the *An. funestus* and *An. gambiae* complexes (RR 2.5) as well as culicine (RR 1.8) mosquitoes than when incandescent or white LED lights were used (Fig. 3, Table 2). There was no significant interaction between the trap type and the quality of light. Outdoor mosquito density estimates were similar across both trap types and quality of light (Table 2).

**Table 2:** Association between trap type, light quality and mosquito catch during field evaluation

	Means (95% CI)	Rate ratio (95% CI)	p-value	Mean (95% CI)	Rate ratio (95% CI)	p-value
	Indoors			Outdoors		
	*An. funestus s.l*					
Trap type						
CDC	8.3 (5.8–11.7)	1		2.2 (1.4–3.3)	1	
SB	9.1 (6.0–13.9)	1.1 (0.8–1.6)	0.709	3.6 (2.1–6.2)	1.6 (0.8–3.3)	0.229
Light quality						
White	8.7 (6.1–12.3)	1		2.3 (1.5–3.4)	1	
UV	21.8 (17.5–27.1)	2.5 (1.7–3.8)	< 0.001	3.6 (2.0–6.2)	1.6 (0.8–3.1)	0.294
	*An. gambiae s.l*					
Trap type						
CDC	5.6 (3.9–8.0)	1		1.5 (1.0–2.2)	1	
SB	5.3 (3.8–7.4)	0.9 (0.6–1.3)	0.811	2.5 (1.5–4.1)	1.7 (0.9–3.1)	0.126
Light quality						
White	5.4 (4.0–7.3)	1		1.7 (1.2–2.4)	1	
UV	13.7 (10.9–17.1)	2.5 (1.7–3.6)	< 0.001	2.3 (1.4–3.9)	1.4 (0.7–2.6)	0.346
	Other *Anopheles*					
Trap type						
CDC	0.2 (0.1–0.4)	1		2.3 (1.7–3.1)	1	
SB	0.2 (0.1–0.6)	0.4 (0.1–2.4)	0.619	1.8 (1.1–2.9)	0.8 (0.4–1.4)	0.351
Light quality						
White	0.2 (0.1–0.4)	1		1.9 (1.2–3.1)	1	
UV	0.4 (0.2–0.7)	0.6 (0.1–2.9)	0.281	2.1 (1.5–2.9)	1.1 (0.6–1.9)	0.887
	*Culicines* (*Culex, Aedes, Mansonia*)					
Trap type						
CDC	5.7 (4.7–7.0)	1		12.0 (8.3–17.3)	1	
SB	5.8 (4.4–7.7)	1.0 (0.7–1.4)	0.807	11.9 (7.9–18.1)	0.9 (0.6–1.7)	0.963
Light quality						
White	4.1 (3.0–5.5)	1		12.3 (7.8–19.3)	1	
UV	7.5 (6.2–9.0)	1.8 (1.3–2.6)	< 0.001	11.6 (8.5–15.9)	0.9 (0.5–1.6)	0.773

Mosquito collection was done over 64 trap nights comprising 16 trap nights indoors and 16 trap nights outdoors for each trap

Whilst only few male *Anopheles* were trapped in total, majority of them (68.9%, n = 171/248) were collected indoors. Likewise, a greater number of culicines were caught indoors (88.4%, n = 479/542) than outdoors. Indoors, traps with UV light were more efficient in trapping male mosquitoes, catching twice as many male *Anopheles* (RR 2.2, 95% CI 1.2–4.1) and culicines (RR 2.3, 95% CI 1.5–3.5) than those with white light. Outdoor catches of male *Anopheles* and culicines were similar in traps with UV and white lights. The trap type did not affect the abundance of mosquitoes collected.

Both trap types also contained a number of non-target insects such as Lepidoptera (moths), Coleoptera (beetles), and Diptera (houseflies, midges). Majority of them were trapped outdoors (91.3%, n = 527/577) with similar efficiency for both trap types. Contrary to the mosquito collections, there was a strong association between UV light and the abundance of these non-target insects outdoors (RR 3.0, 95% CI 2.0–4.5). However, in the indoor environment, the catches of non-target insects were similar in traps with UV and white light.

### Property of light emitted by different traps

**Fig. 4:**
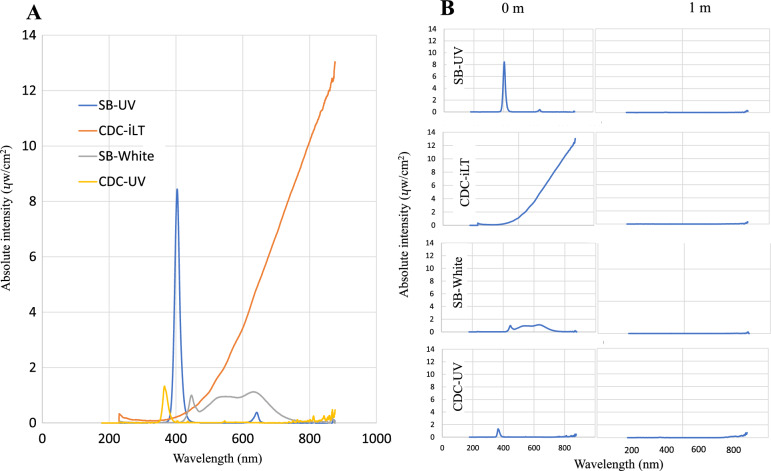
Absolute spectral wavelength and intensity of light emitted by traps **A** at source and **B** 1 m away from the trap

The SB-UV emitted light at a wavelength between 376 and 448 nm (peak, 406 nm) and the CDC-UV at a wavelength between 344 and 407 nm (peak, 365 nm), both within the longwave ultraviolet light spectrum bordering the visible (for human beings) light (near-UV; [49]). Light emitted from the incandescent bulb used in the CDC-iLT was partly in the visible spectrum but the highest intensity of it was in the infrared range of 750–900 nm (Fig. 4), a wavelength that is invisible to mosquitoes [50]. The white LED from the SB trap emitted very low intensity light across a wide range from 485 to 700 nm (Fig. 4).

Notably, the light emitted from the traps was only detectable at closest proximity from the source of light. Already at one metre distance from the source, the UV intensity was nearly completely diminished. The only spectral wavelength still measured was in the infrared (Fig. 4).

## Discussion

The overall aim of this study was to assess if the SB trap might be a possible substitute for the CDC light trap which is a standard trap widely used for vector research and recommended for surveillance across sub-Saharan Africa [51, 52]. Results here show that both traps collect vectors in similar numbers when used in the same field site. Also, both traps perform comparably in the indoor or outdoor environments, hence, confirming that the SB trap could be an alternative trapping device in mosquito surveillance programmes.

Routine entomological surveys in disease vector control programmes are a prerequisite for evidence-based decision-making, and yet, many malaria endemic countries do not implement these at scale [53, 54]. In addition to effectiveness in catching mosquitoes, factors such as cost, ease of use and portability are important in considering the deployment of traps for mosquito surveillance [55]. Whilst not the leading cause, a contributing factor is the difficulty in accessing and maintaining traps and batteries. Costs and logistics affect scalability and can hinder the implementation of surveillance programs [55–57]. The SB trap is robustly built of polyvinyl chloride which makes it sturdy [34] and thus suitable for use in harsh environments in rural sub-Saharan Africa. The solar panel on top of the trap can be used to charge the trap’s built-in/integrated battery during the day to prepare for the next trap night [34] making the trap independent from unreliable power grids in many rural settings in sub-Saharan Africa [58]. This flexibility to charge the SB trap’s built-in battery with three alternative methods using solar energy, mains electricity or through vehicle auxiliary power cable allows it to fit in varying field circumstances. The lower per unit cost of the SB trap than the CDC trap could also make this trap an attractive alternative for mosquito sampling. In the project, traps were purchased in October 2020 for USD165.90 a unit for the SB traps and for USD188.90 for a CDC-UV trap. Furthermore, the delivery time from the manufacturer to the field site in Kenya as well as the shipping costs were over two times higher for the CDC light traps from USA than the Silver Bullet traps from South Africa.

The SB trap’s strong built and sturdiness, can however, also be a limitation for operational use under certain circumstances since the trap cannot be disassembled for transport between field sites and has a considerable weight. Project field staff using motor bikes or bicycles for transporting traps between collection points highlighted the difficulty to travel with several traps. Thus, although the SB trap offers a reasonable alternative for mosquito surveillance its bulkiness could also limit the use in routine mosquito surveillance especially in locations with limited motorized transport. Further modifications by the manufacturer might be warranted to ease operations, should the trap be used at large scale.

The quality of light, not the trap type, was strongly associated with the efficiency of trapping mosquitoes in the indoor environment. In the relatively confined space of a rural house and in the presence of a human blood host sleeping under a mosquito net, traps emitting light in the UV spectrum trapped twice as many mosquitoes of all genera, and both females and males, than traps emitting white LED or incandescent light. The superiority of UV light was also observed under experimental conditions in the large field cages. The greater spectral sensitivity of nocturnal mosquitoes towards light in the UV spectrum and the use of UV light for orientation in general and towards traps has been well described [27, 59, 60], yet UV light traps are rarely used for routine vector surveillance. The reason for this might be because the UV light catches a higher density of non-target insects when used in traps [61, 62] making sorting time-consuming and difficult. However, the results from the field evaluation suggest that trapping non-target insects in large numbers is only a problem in the outdoor environment, where UV light was not advantageous over other light sources, but not indoors where UV light resulted in significant higher catches of mosquitoes.

In the outdoor environment, the quality of light was not associated with the trapped mosquito numbers and trap catches were generally low. Spectrometry indicated that the light from any of the traps was only measurable in a dark room at very close encounter, whilst the detection of emitted light in all traps was drastically reduced already at one metre distance. It appears plausible to assume that in the confined indoor space of a rural African house and in close proximity to the blood host, mosquitoes will eventually get into the range of the light and get trapped. However, this is unlikely in the wide open outdoor environment, where the cues released from the trap need to compete with natural cues such as other light sources including lunar light [63], and attractive natural blood hosts releasing a multitude of chemical odours and heat. The estimated outdoor abundance based on any of the light traps tested was likely an underestimate of the actual number of malaria vectors and other mosquitoes in the study areas. Previous studies comparing CDC light traps and human landing catches for mosquito sampling observed substantially reduced catches in light traps, and thus concluded that the traps are not appropriate to use in estimating the density of outdoor mosquitoes [64, 65]. In another study, Njoroge et al*.* [66] showed cattle-baited traps to catch a significantly higher number of *Anopheles* and culicines in western Kenya, than would be expected if light traps were used [25, 67].

Monitoring changes in the outdoor *Anopheles* composition and densities is important due to the increasing importance of residual malaria transmission [68, 69]. Several outdoor mosquito sampling tools such as resting boxes, clay pots, pit shelters, furvela tent trap and host decoy trap have been evaluated under different epidemiological settings with varying degrees of success [70–75]. However, each of these tools have their benefits and drawbacks. While pit shelters effectively provide attractive microhabitat for resting of mosquitoes [76], the stationary nature of the pits is a major hindrance for operational large-scale deployment [22]. Similarly, while clay pots are small and portable for large-scale deployment, retrieving mosquitoes by aspiration may result to collection bias due to variation in skills among collectors [22]. Recent studies have demonstrated tent traps such as furvela trap as efficient in sampling *Anopheles* when used outdoors [73, 75], however, there are concerns whether the trap truly samples outdoor mosquitoes or is an imitation of an indoor CDC trap [73]. Moreover, the potential exposure of human occupants in tent traps to mosquito bites is a concern in their routine use for mosquito surveillance [75]. Altogether, these observations highlight the need for more robust tools to collect mosquitoes in the outdoor environment.

The general limitation of light traps in attracting representative numbers of mosquitoes in an open environment was also demonstrated by the results from the semi-field experiments. Even though traps were baited with light and CO_2_, a single trap in a system only recaptured a quarter of the mosquitoes released during any given trap night. This contrasts with human landing collections implemented in the same systems, which resulted consistently in 80% recovery of the released mosquitoes [77, 78]. This supports the conclusion that in open outdoor spaces, where concentrations of odour cues are quickly diluted due to prevailing climatic conditions [79, 80], light traps have severe limitations and surveillance data needs to be interpreted with caution [81]. These semi-field experiments, where two traps were presented in a competing set-up, illustrated the importance of combining essential cues mimicking a blood host for attraction of host-seeking mosquitoes. When the CDC-iLT and SB-White trap were placed in the same system, the trap with the incandescent light source was far more attractive to host-seeking female *Anopheles* than the trap with a white LED light. Both the incandescent bulb and white LED emit light in the spectrum visible for human beings which is not visible to nocturnal mosquitoes [50, 82]. However, incandescent bulbs, unlike LEDs, generate light through heat (infrared radiation) and heat in turn has been shown to be an important host cue for which host-seeking *Anopheles* females have specialized sensors located at their antennas [83]. The combination of this thermal radiation with the CO_2_ supplied with all traps was likely closer to an expected host cue than CO_2_ alone, given that the white LED light was largely invisible to the host-seeking mosquito. This is in line with other work on trap development, that clearly demonstrated the benefit of combined odour and visual stimuli with a thermal signature [84]. Recent work also showed that CO_2_ can induce a strong attraction to specific spectral bands at higher wavelength in the red spectrum which is emitted by human skin [85], and is emitted at higher intensity by incandescent bulbs than white LED light. LEDs are now widely used in mosquito traps since they are more energy efficient and have a longer lifespan [13, 86]. Based on the results from this study, it can be assumed that the CDC light trap with LEDs would be equally attractive as the SB traps with LEDs. Interestingly, however, odour cues combined with light emitted in the UV spectrum, which is highly visible for nocturnal mosquitoes, was the most attractive combination of cues in our semi-field experiments without any heat source. This increased attraction of the combination of odour cues with UV light was also observed in the field experiments where heat, body odour and CO_2_ was provided by the human bait protected by a bed net. The interaction of UV light with other host and environmental cues might warrant further studies.

## Conclusions

The Silver Bullet light trap was equally efficient as the CDC light trap in catching *Anopheles* and culicine mosquitoes under field conditions. It might be desirable to conduct additional studies to assess the performance of the trap in other ecological settings to confirm these findings. The use of LEDs emitting light in the UV spectrum should be considered for indoor vector monitoring to improve trapping efficiencies especially in settings or seasons with low mosquito abundance. The results also support previous work [84, 87] outlining the low efficiency of light traps in the outdoor environment and the need to develop simple to use and scale, yet more attractive and hence competitive trapping devices combining visual, chemical and potentially thermal cues.

## Data Availability

All data will be made available on reasonable request to the corresponding author.

## Abbreviations

CDC: Centres for Disease Control and Prevention
SB: Silver Bullet
LEDs: Light emitting diodes
*An*: *Anopheles*
PCR: Polymerase chain reaction
CO_2_: Carbon dioxide
UV: Ultraviolet
ILT: Incandescent light trap
